# Diversity of Extracellular Vesicles in Human Follicular Fluid: Morphological Analysis and Quantification

**DOI:** 10.3390/ijms231911676

**Published:** 2022-10-02

**Authors:** Anne-Sophie Neyroud, Regina Maria Chiechio, Gregory Moulin, Solène Ducarre, Claire Heichette, Aurélien Dupont, Mathieu Budzynski, Pascale Even-Hernandez, Maria Jose Lo Faro, Marina Yefimova, Valérie Marchi, Célia Ravel

**Affiliations:** 1 CHU Rennes, Service de Biologie de la Reproduction-CECOS, 35000 Rennes, France; 2 Environnement et Travail (IRSET)—UMR_S 1085, Institut de Recherche en Santé, F-35000 Rennes, France; 3 CNRS UMR 6226, Institut des Sciences Chimiques de Rennes, Université Rennes 1, Avenue du Général Leclerc, 35042 Rennes, France; 4 Dipartimento di Fisica e Astronomia “Ettore Majorana”, Università Di Catania, Via Santa Sofia 64, 95123 Catania, Italy; 5 UMS 3480, US_S 018, BIOSIT, Univ. Rennes, CNRS, Inserm, 35000 Rennes, France

**Keywords:** extracellular vesicles, follicular fluid, human, oocyte, morphology

## Abstract

The oocyte microenvironment constituted by the follicular fluid (FF) is a key for the optimal development of female gametes. Its composition reflects the physiological state of the ovarian follicle. The particularity of FF is to contain a huge diversity of extracellular vesicles specific to women, in the same way as seminal plasma in men. Here, we described and compared morphological aspects of broad subcategories of human FF-related Extracellular Vesicles (EVs). EVs participate in physiological and pathological processes and have potential applications in diagnostics or therapeutics. EVs isolated from FF are involved in different biological functions related to follicular growth, oocyte maturation, and embryo development. However, knowledge on the morphology of FF-derived EVs is limited, mainly due to their sub-micrometer size and to intrinsic limitations in methods applied for their characterization. The aim of this study was to provide a comprehensive morphological description of EVs from FF of healthy subjects and quantification. EVs separation was realized by centrifugation, with comparison of the EV yield obtained from differential centrifugation and one-step ultracentrifugation. Cryo-Transmission Electron Microscopy was used to reveal the morphology, size, and phenotype of EVs. Dynamic Light Scattering (DLS) and Nanoparticle Tracking Analysis (NTA) were used to quantify and analyze the size distribution for each centrifugation step. We performed a comprehensive inventory of human follicular fluid EVs. We show that human FF contains a huge diversity of EVs. This study brings novel insights on EVs from normal FF and provides a reference for further studies of EVs in ovarian diseases.

## 1. Introduction

The ovarian follicle represents a reproductive unit consisting of an oocyte surrounded by many somatic cells composed of cumulus granulosa, mural granulosa, theca cells and follicular fluid (FF). FF formation is due to the infiltration of several plasma components by transudation. It provides nutrition for the oocyte and allows it to mature inside the follicle [1] Follicle growth and oocyte maturation are connected by a constant exchange of signals between somatic cells and germ cells. Granulosa cells as ovarian somatic cells may interact with each other or with the oocyte via intercellular communications and homeostatic phagocytosis [2]. The cross-talk between the oocyte and granulosa cells occurs with gap-junctions but in the last decade, extracellular vesicles (EVs) were identified in follicular fluid as a novel mode of communication in the ovarian follicle [3,4]. EV is the generic term for particles naturally released from the cell that are delimited by a lipid bilayer without any functional nucleus [5]. Isolated EVs from FF have been shown to affect the expression of a select few genes in cultured granulosa cells by regulating genes involved in follicular development, meiotic resumption, and ovulation [6]. Changes in EV biogenesis or uptake occur during antral follicle development [7]. As important environmental sensors, EVs and their cargo respond to environmental toxins [8].

Isolating EVs from FF where samples are biobanked before research use is challenging. Several methods are used to isolate EVs. The most commonly used method is UltraCentrifugation (UC) in the field [9]. UC uses centrifugal force to separate and purify EVs by using high centrifugal speed for sufficient time for individual EVs to travel the length of the tube into a pellet while being less efficient at pelleting particles. Different commercial EV isolation kits, or commercial EV isolation kits compared to size exclusion columns or ultracentrifugation, are used for small volumes while density gradients are used for large volume EV isolations. The sequential use of two or more isolation methods greatly improved depletion of lipoprotein and protein contaminants; however, this was correlated with a significant decrease in overall EVs number [10]. Exosomes are defined by their content of endosome-associated proteins including tetraspanins CD9, CD63, and CD81. The CD63 EV marker is mostly used to correlate the amount of protein in the sample but not to the NTA particle counts. Flow cytometry detection of CD63 is an alternative approach to confirm EVs [10]. The presence of EVs by CD63-positive staining in ovarian follicular fluid was first identified in horses [3].

Diverse roles are assigned to FF-derived EVs described to play a crucial role in intercellular communication within the ovarian follicle [11]. FF provides a favorable environment for the normal development of a competent oocyte to be fertilized and undergo embryogenesis. FF-derived EVs are involved in the regulation of pathways controlling follicular growth and hormone response as well as oocyte cytoplasmic maturation and meiosis resumption. The supplementation of culture media with EVs isolated from the follicular fluid during oocyte maturation and early embryo development can partially modify metabolic and developmental-related genes as well as miRNA, global DNA methylation and hydroxymethylation [6]. Labeled EVs have been identified in the transzonal projections formed by cytoplasmic extensions of cumulus cells [6] suggesting that there is secretion of these cell-secreted vesicles into the perivitelline space [12]. These vesicles may mediate the transport of information from follicular fluid to the oocyte showing a positive effect on correct embryo development [6]. Indeed, FF-derived EVs are able to induce cumulus–oocyte complex expansion by modulating the expression of genes with known functions in this process, such as prostaglandin-endoperoxide synthase 2 (*Ptgs2*), pentraxin-related protein 3 (*Ptx3*), and tumor necrosis factor alpha-induced protein 6 (*Tnfaip6*) [11]. Moreover, there is the communication between the FF-derived EVs and granulosa cells. Indeed, FF-derived EVs can be taken up by cultured granulosa cells highlighting cell-to-cell communication within the antral follicle [13]. The transfer of bioactive material through the uptake of microvesicles and exosomes from the follicular fluid by surrounding granulosa cells has been demonstrated as well [3].

The inventory of EVs in FF is of great importance for both fundamental knowledge and practical application in the human reproduction field. There is a growing research interest in the characterization of EVs from FF to improve cumulus expansion and oocyte competence to fertilization. Purification methods for EVs can exert a different functional impact on oocyte maturation and subsequent embryo development, showing that EVs from in vivo derived FF exert a positive impact on embryo development [14]. The inventory of FF-derived EVs is important for developing future experimental approaches and to interpret previously published data to understand the role of EVs for human fertility. Therefore, it becomes possible to detect specific signature by analyzing the change in size and morphology distributions of these FF-derived EVs.

The aim of this study was to provide a comprehensive description of EVs from the FF of healthy subjects. We compared the EV yield obtained from differential centrifugation and one-step ultracentrifugation. We did not focus on a single type of exosome because of the high polydispersity of the FF-derived EVs. The use of a commercial EV isolation kit would have limited the analysis to a single type of exosome, whereas we aim for an exhaustive analysis of all the EVs present in the samples. We used Cryo-Transmission Electron Microscopy (Cryo-TEM), which preserves membranes in a close to native state to reveal the morphology and size of EVs. We also used Dynamic Light Scattering (DLS) and Nanoparticle Tracking Analysis (NTA) to measure their size and to quantify them as recommended by recent guidelines [15]. Following a protocol of differential centrifugation to document specific FF-derived EVs morphology [5], we quantified each type of structure in terms of size and morphology.

## 2. Results

### 2.1. Size Separation of EVs by Sequential Centrifugation or Direct Ultracentrifugation

**Figure 1:**
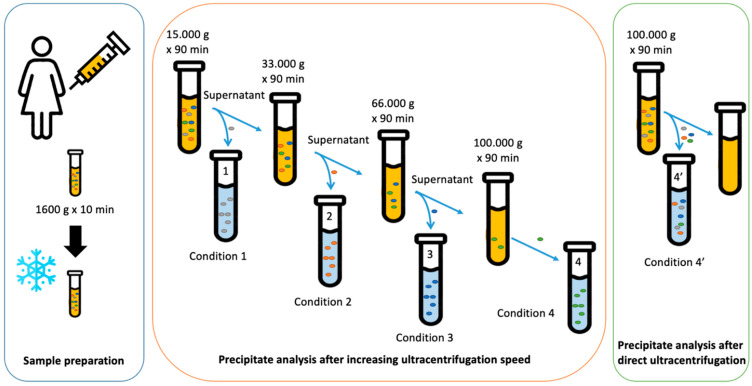
Schematic view of the protocols to separate the different subclasses of EVs in FF.

We proceeded with an increasing speed centrifugation, sequenced in four steps compared to one-step ultracentrifugation of the FF sample. We proceeded with multi-step differential ultracentrifugation, transferring the supernatant into a new tube each time (Figure 1). A first centrifugation at medium speed (condition 1), was used to collect large EVs and large apoptotic bodies, then a centrifugation at intermediate speed (condition 2) to collect a pellet enriched in medium-size EVs and aggregates of small EV supernatant and finally two centrifugations at higher speed (condition 3 and condition 4) to collect a pellet enriched in small EVs. A direct ultracentrifugation at the highest speed (as condition 4) over the FF sample was performed (condition 4′) to obtain the entire EV-FF population.

The NTA technique permits to evaluate the particle concentration and diameter under each condition.

**Figure 2:**
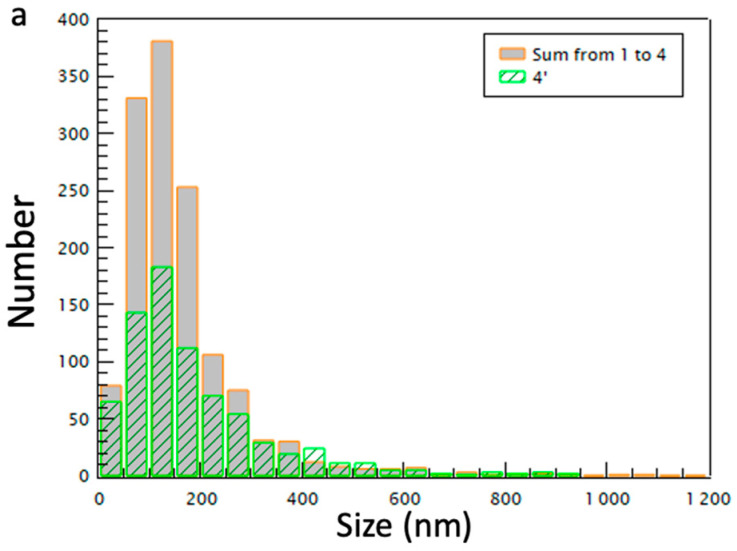
Size histograms of the EV populations obtained from the analysis of Cryo-TEM images using sequential centrifugation (sum of conditions 1–4, in orange) and one-step ultracentrifugation (condition 4′, in green).

The sum of the NTA concentrations of the four conditions (1–4) (1.6 × 10^11^ particles/mL) is found to be higher than that from the condition 4′ (1.11 × 10^11^ particles/mL) (see Table 1). This can be explained because the EV population of the one-step ultracentrifugation, condition 4′, does not correspond to the sum of the four conditions since the total time used to perform the four centrifugations (300 min) is much greater than that used for the condition 4′ (90 min); therefore, a part of the EVs remained in the supernatant. There was a significant decrease in the number of vesicles sized between 50 and 200 nm following one-step ultracentrifugation compared to the sum of conditions 1–4 as shown in Cryo-TEM images (Figure 2). Even at a high speed, not all the smaller vesicles appear to be separated from the supernatant. This observation is attributed to the time of the experiment in the condition 4′ which is much shorter than the time of the sum of conditions from 1 to 4.

**Figure 3:**
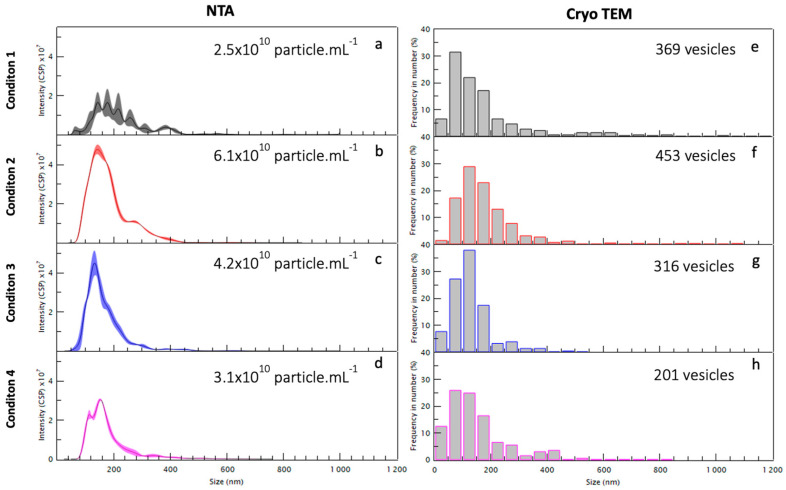
Size histograms of the EVs populations obtained from analysis of NTA (**a**–**d**) and Cryo-TEM (**e**–**h**) images using sequential centrifugation 1, 2, 3, and 4.

As summarized in Table 1 and Figure 3, the first centrifugation, condition 1 (15,000 *g* for 90 min) collected 2.5 × 10^10^ particles/mL enriched in large EVs and large apoptotic bodies with dimensions above 200 nm, in particular between 200 and 300 nm with some large aggregates of 500–700 nm and some small EVs of 50 nm (see Figure 3a). The centrifugation at intermediate speed, condition 2 (33,000 *g* for 90 min), collected 6.1 × 10^10^ particles/mL and the size distribution is between 50 and 300 nm, without any bigger EVs (see Figure 3b). The centrifugation at higher speed, condition 3 (66,000 *g* for 90 min) (see Figure 3c) and condition 4 (100,000 *g* for 90 min) collected, respectively, 4.2 × 10^10^ particles/mL and 3.1 × 10^10^ particles/mL, both enriched in small EVs of similar sizes (below 200 nm) (see Figure 3d). The one-step ultracentrifugation, condition 4′ (100,000 *g* for 90 min) collected 1.1 × 10^11^ particles/mL.

### 2.2. Morphology and Size Analysis of the EV Subpopulations

#### Different Subcategories of Extracellular Particles and Vesicles Found in Human FF

All the particles visualized using Cryo-TEM were classified according to their morphology. The size of these particles varies from 5 to 700 nm (see Figure 3e–h). Preparations at each centrifugation stage analyzed showed a population of small extracellular vesicles (30–100 nm) resembling exosomes and a population of large extracellular vesicles (>100 nm) resembling microvesicles. Conversely to seminal plasma [16], we did not find any myelinosome in this experiment (200–700 nm). The NTA counted an average of 4 × 10^10^ particles/mL. A total of 420 images were analyzed. For each centrifugation step, 55 images from condition 1 (15,000 *g*), 54 images from condition 2 (33,000 *g*), 50 images from condition 3 (66,000 *g*), and 246 images from condition 4 (100,000 *g*) were analyzed. In total, 188 images corresponding to the most informative condition 4′ were analyzed.

**Figure 4:**
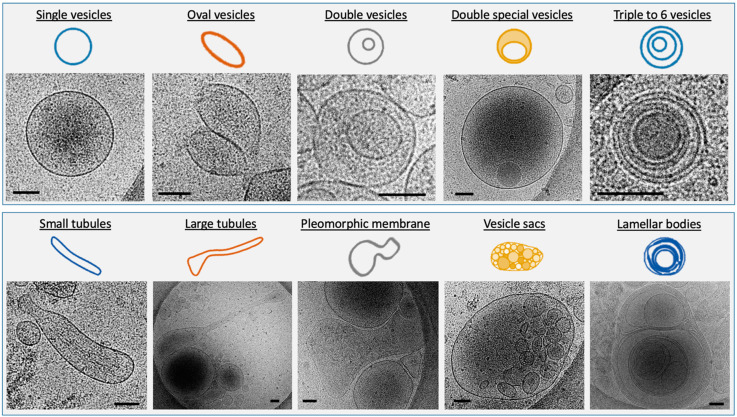
Cryo-TEM images of the 10 subcategories of EVs depending on their size and their morphology. Scale bar: 100 nm.

All the structures completely contained within the images were measured and divided into 10 subcategories according to Höög et Lötvall’s classification [17] depending on their size and their morphology (Figure 4). We added the quantification of incomplete structures, probably damaged. Single vesicles have a 5 nm-thick bilayer and are the most prevalent EVs. We distinguished oval vesicles as a shape reminiscent of the single vesicles. Double vesicles contain a smaller vesicle inside of a larger vesicle; double special vesicles have a larger diameter than the double vesicles. Larger EVs contain often electron-dense material as cargo. Triple to six vesicles consist of two small vesicles stuck into a larger one. Some are more complex arrangements of one larger vesicle containing smaller vesicles. Tubules present a ratio between length and width larger than 5 and some contained filaments. Pleomorphic membrane structures include pear-shaped membrane compartments. Vesicle sacs are several vesicles arranged inside a larger membrane. Vesicles with double-membrane bilayers were also seen inside vesicle sacs. Large and complex multilayered membrane structures visualized in the FF samples were described as lamellar bodies.

**Figure 5:**
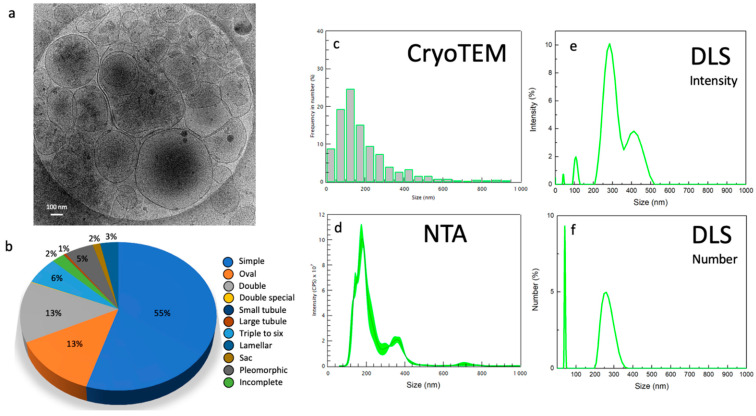
(**a**) Cryo-TEM images and (**b**) morphological analysis of FF-EVs after high speed (100,000 *g*) centrifugation without differential steps (condition 4′). Corresponding size histograms for the same sample obtained from Cryo-TEM (**c**), NTA (**d**), and DLS (**e**,**f**) showing intensity and number.

To get around the problem of size dispersity and develop a complete description of the EV population using fast techniques, for each centrifugation condition (1–4 and 4′) described in Figure 1, the EVs size distribution was determined using various characterization techniques and compared considering the advantages and disadvantages of each technique. The images were analyzed in cryo-TEM and compared to both NTA and DLS. As shown in Figure 5, the condition 4′ gives an overall idea about the diversity in size of the whole population. To compare EVs number belonging to the different conditions via Cryo-TEM images, a similar number of photos were analyzed for each centrifugation condition. Samples were analyzed using NTA to evaluate the EVs concentration and size distribution. Since the sample has a high polydispersity, it is difficult to detect EVs using NTA in the case of a hydrodynamic radius smaller than 100 nm. Therefore, all the EVs with a smaller radius were not considered, underestimating the concentration of the EVs.

**Figure 6:**
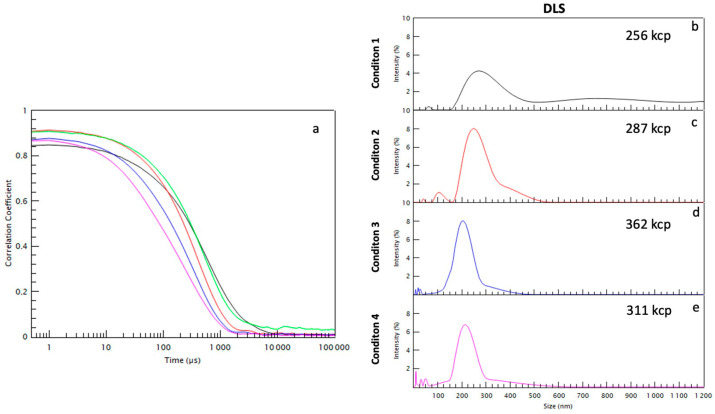
(**a**) Dynamic light scattering correlograms for the different conditions and (**b**–**e**) the corresponding size histogram in intensity for the differential centrifugation conditions (1 to 4).

The different conditions were analyzed with DLS to examine the EVs with a diameter smaller than 100 nm. This DLS technique does not allow to quantify the number of particles/mL as NTA, but it is able to provide an estimation of the ratio between the different EVs families. Through the DLS intensity measurements, there is an overestimation of large compared to small EVs; for this reason, the number graph was also presented which enhances the signal coming from small EVs that have a lower scattering signal. In the case of the condition 4′, two main EVs subpopulations are visible using NTA and DLS around 100–200 and 300–400 nm, respectively. One can also notice the appearance of larger EVs around 500–700 nm visible using NTA. Finally, an abundant subpopulation of very small EVs around 10–30 nm is clearly systematically detected using DLS through a percentage number analysis (see Figure 6).

**Figure 7:**
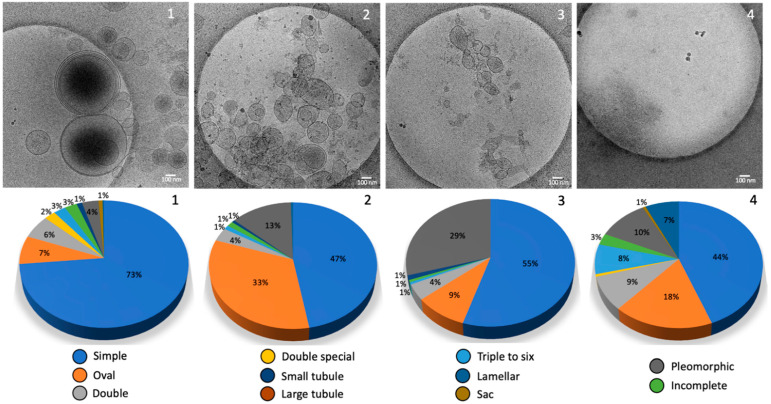
Morphology of the different conditions analyzed using Cryo-TEM: images used for morphological quantification pie histogram and corresponding pie histograms and vesicle percentages according to the morphological classification in Figure 4.

Finally, the Cryo-TEM technique permits to examine the precise morphology of the different-sized EVs subpopulations as shown in Figure 7.

The samples resulting from sequential centrifugation were analyzed through the same complementary techniques. As shown in Figure 3 and Figure 7, the condition 1 is enriched in large EVs and large apoptotic bodies with dimensions above 200 nm, in particular between 200 and 300 nm with some large aggregates of 500–700 nm and some small EVs of 50 nm as shown in Figure 3. The condition 2 has the largest concentration of EVs using NTA compared to the other conditions and the size distribution is between 50 and 300 nm, without the bigger EVs (Figure 3 and Figure 7). Conditions 3 and 4 can be combined since they present a very similar population enriched in small EVs of similar sizes (below 200 nm) (Figure 3 and Figure 7). Through DLS, particles with a radius of about 10 nm were found (Figure 6) and they represent the majority of the sample, which is confirmed in the Cryo-TEM images, despite the very small dimensions making them difficult to identify and count through Cryo-TEM.

## 3. Discussion

The presence of EVs in FF from human, bovine, and equine sources has been noticed and termed folliculosomes [18]. In our study, a complete morphological and size inventory of these folliculosomes was made for the first time. This inventory of extracellular structures in follicular fluids reveals a very diverse environment where single vesicles are predominant with an enrichment in dark ones with high-speed ultracentrifugation. In total, 10 different relatively distinct morphological categories are described according to Hoog and Lotvall’s classification [17]: vesicle sacs, lamellar vesicles, pleomorphic membrane structures, multi-vesicles, oval vesicles, single vesicles, double vesicles, double special vesicles, large tubular vesicles, and small tubular vesicles. Of note, we often observed an electron-dense core inside double vesicles, double special vesicles, triple or more vesicles.

The separation of EVs still represents a critical step and methods to isolate EVs are currently highly diverse. Depending on which separation method is employed, the results can be different from the same sample [19]. Results of the isolation and purification of exosome preparations obtained in different laboratories are comparable when their centrifugation protocols are correlated through “cut-off-size”-based equalization [20] Here, isolation was achieved by comparing yield and selection according direct or differential ultracentrifugation. Differential ultracentrifugation can isolate relatively pure populations of exosomes and is generally considered to be the gold standard technique for exosomal isolation [21]. If direct ultracentrifugation seems to lose yield, it seems to obtain more large structures. A mechanism of membrane fusion could arise during the manipulation of fluids [22]. On the other hand, the differential ultracentrifugation, if it allows a better yield, seems to be less efficient for quantifying the large structures. This aspect may be due to the presence of aggregates at each stage, which are difficult to resuspend. Moreover, it is unclear what effect pelleting a fluid membrane structure against a solid surface at high g forces for prolonged time periods has on membrane integrity and vesicle content [23].

So far, the Cryo-TEM morphology of EVs present in human FF has not been studied. We show here the extreme diversity of extracellular structures in human follicular fluid. Remarkably the EVs content of FF strikingly resembled those previously described for seminal plasma in men. Our data show that like other human fluids, FF contains spherical EVs about 200 nm in diameter, tubular EVs and large fragments as described previously [24]. FF also contains microvesicles ranging from 100 to 1000 nm, which are formed by budding from the plasma membrane; the exosomes having a diameter of 30–150 nm formed by inward budding of the late endosome lumen which forms a multivesicular body (MVB), that are secreted by fusion of MVB with the plasma membrane. Membrane vesicles are limited by a lipid membrane, as evidenced by the presence at their periphery of two dark lines about 5 nm apart, which is a characteristic feature of lipid bilayers by Cryo-TEM. Compared to Cryo-TEM, NTA overestimates the amount of EVs due to the presence of lipoproteins and protein aggregates and highlights a limitation when dealing with human fluid as described by others [10].

Vesicles are defined as round structures carrying a lipid bilayer. This criterion is very discriminative and the numerous small electron dense particles without any visible lipid bilayer must not considered as true EVs. Centrifugation and freezing processes may induce physical strengths modifying the EV structure. However, samples are frozen so quickly that there is no osmotic stress. The oval or tubular shape indicates an excess of lipid membrane surface or a volume loss with an osmotic pressure higher outside than inside. Membrane-shaping proteins, such as BAR-domain proteins, might influence the shape of the oval vesicles and tubules [17]. We chose the most commonly used separation technique but ultracentrifugation can cause aggregation of EVs, which might lead to artifacts during analysis.

Basically, the extracellular environment of human FF was found to be as diverse as seminal plasma. However, if three subtypes of EVs have been described in the male genital tract, microvesicles, myelinosomes, and exosomes [25], we did not find any myelinosome in our samples, but only microvesicles and exosomes. Obviously, it does not mean that there are none and other analyses will be necessary before confirming the absence of these very particular structures in human FF. Our data show that FF-derived EVs mainly consist of five major subcategories of EVs and five subcategories of extracellular membrane compartments including lamellar bodies. In addition, small particles of 10–50 nm in size that were not surrounded by a lipid bilayer were also observed in FF samples. They likely consisted of protein or lipoprotein complexes. Similar to blood plasma, FF contained the pleomorphic membrane compartments and tubular structures [24].

It has been suggested that biological molecules, such as lipid rafts or cytoskeletal components might modify the shape of EVs [17,26]. One structure of particular interest is the vesicle sac as it harbors EVs of different morphologies within the same membrane. The double bilayer may be indicative of an organelle source such as REG or endosome. It is worth noting that large EVs (>400 nm) and very small EVs (<50 nm) are not well quantified by NTA [5].

According to Figure 5a, several EVs present a density aspect suggesting an important content. The best known cargo of EVs is the packaging of miRNAs [7]. FF-derived EVs contains miRNAs, some of them playing significant roles in steroidogenesis. Santonocito et al. isolated and characterized exosomes from human FF using ultracentrifugation, NTA and flow cytometry. They found 32 exosomal miRNAs FF-derived EVs able to regulate follicular development, meiotic resumption, and ovulation [27]. Specific FF miRNA profiles have been associated with oocyte quality and embryo outcome (for review [28]). By investigating the difference in the microRNA profiles of fertile women and polycystic ovary syndrome (PCOS) patients, it has been shown that the concentration of EVs was higher in the FF of PCOS patients indicating signaling disturbances [29]. All these studies highlight important regulatory roles that folliculosomes most likely have. These EVs contain RNAs that can be transferred to the gamete and most likely play a role in the development of the RNA profile during gametogenesis and early embryo development. It is not clear if EVs directly control gene expression, but they may participate in the epigenetic regulation of gene expression by transporting and delivering specific molecules potentially implicated in epigenetic inheritance. This mechanism might be of relevance in the genital tract with the modification of the gamete environment [30].

Our study brings novel insights on EVs from normal FF and provides a reference for further studies of EVs in disease situations. We believe that such characterization using complementary DLS, NTA, and Cryo-TEM techniques to analyze the morphological diversity of the extracellular vesicles could be a useful tool to determine anomalies related to pathologies. Further studies on different molecular cargos of FF-EVs will open new promising applications in diagnosis and therapy. Among them are the discovery of non-invasive markers of different pathologies, the identification of new therapeutic targets, and development of innovative drug-delivery systems [31]. This can open new perspectives for the optimization of assisted reproductive technologies by improving in vitro oocyte maturation in humans.

## 4. Materials and Methods

### 4.1. Patients

A pooled sample (150 mL) was collected from 20 healthy normal-ovulating patients who had undergone intracytoplasmic sperm injection due to a male factor. Samples were provided by the GERMETHEQUE Biobank, dedicated to human fertility. Informed consent was obtained from each couple for the use of the follicular fluid sample that was obtained during oocyte retrieval for the ICSI treatment. Approval CP-GM n° 20,210,808 was obtained for this study.

Patients were stimulated with recombinant FSH. Ultrasound monitoring of the follicular development occurred from day 6 of ovarian stimulation until the day of oocyte retrieval. When at least 3 ovarian follicles had grown up to 18 mm in diameter, 10,000 IU human chorionic gonadotropin was administered, and the follicles were aspirated 36 h later.

The patients’ average age was 31.5 years, the average Antral Follicle Count was 21, and the average AMH (anti-Müllerian hormone) concentration was 3.4 ng/mL.

### 4.2. Sample Preparation for Analysis

FF was collected by transvaginal ultrasound-guided aspiration of follicles up to 18 mm in diameter. Fluid samples of 10 mL were centrifuged at 1300× *g* for 10 min at 4 °C to remove cells and the supernatant was stored at −196 °C degrees. The samples were previously heated at room temperature for 30 min and the FFs belonging to 20 different patients were pooled. Finally, the pooled sample (150 mL) was divided into 10 tubes (15 mL) of FF sample ready for ultracentrifugation and further analysis.

### 4.3. Ultracentrifugation

In total, 8 tubes of 15 mL of pooled FF sample went through multi-step differential ultracentrifugations, using the supernatant from the previous condition: condition 1 (15,000 *g* for 90 min), condition 2 (33,000 *g* for 90 min), condition 3 (66,000 *g* for 90 min), and condition 4 (100,000 *g* for 90 min). Two tubes (15 mL) of pooled FF sample were used for the direct ultracentrifugation performed at 100,000 *g* for 90 min (condition 4′). Each pellet was resuspended in 250 μL of PBS (sodium chloride, 150 mM, and sodium phosphate, 150 mM, pH = 7.2). All steps were performed at 4 °C.

### 4.4. Cryo-Transmission Electron Microscopy (Cryo-TEM)

Vitrification of the FF pooled sample (10 μL) under the different centrifugation conditions (1–4 and 4′) was performed using an automatic plunge freezer (EM GP, Leica) under controlled humidity and temperature [32]. The samples were deposited to glow-discharged electron microscope grids followed by blotting and vitrification by rapid freezing into liquid ethane. Grids were transferred to a single-axis cryo-holder (model 626, Gatan) and were observed using a 200 kV electron microscope (Tecnai G2 T20 Sphera, FEI) equipped with a 4 k × 4 k CCD camera (TemCam-XF-416, TVIPS). Micrographs were acquired under low electron doses using the camera in binning mode 1 and at a nominal magnification of 25,000×. These images were used for morphological and size analysis.

### 4.5. Dynamic Light Scattering (DLS)

Each sample (pellets re-suspended in 250 μL PBS buffer) was diluted 100 times with PBS just before measurement, to avoid structural modification linked to the dilution process. The measurements of the mean hydrodynamic diameters were performed at an angle of 173° using a Nanosizer ZEN3600 (Malvern Instruments, England) and collected at 25 °C.

### 4.6. Nanoparticle Tracking Analysis (NTA)

Each pellet was resuspended in 250 μL of phosphate-buffered saline (PBS). For NTA, each sample was diluted just before measurement with PBS following the manufacturer’s instructions. The initial dilution factor used was 10 in PBS for samples 1, 2, 3, and 4 and 100 for sample 4′. Samples (700 μL) were injected into the NanoSightLM10 unit (Malvern Instruments, Malvern, UK) with a 1 mL sterile syringe. Capture and analysis settings were manually set according to the protocol. Using the NanoSight LM10 instrument, vesicles were visualized using laser light scattering, and the Brownian motion of these vesicles was captured on video. The number of tracks always exceeded 200, and three size distribution measurements were taken for each sample. Recorded videos were then analyzed with the software NanoSight NTA 3.1 software (Malvern, UK) which provided high-resolution particle size distribution profiles and concentration measurements of the vesicles in solution. EV analysis was performed in three videos of 30 s each at 25 °C.

## Figures and Tables

**Table 1:** Evaluation of the particle concentrations using NTA and Cryo-TEM techniques. The distribution in diameter is provided as a range of mean size. Each centrifugation condition was performed during 90 min.

Condition Centrifugation Speed	1 15,000 *g*	2 33,000 *g*	3 66,000 *g*	4 100,000 *g*	4′ 100,000 *g*
**NTA** (particle·mL^−1^) mean size (nm)	2.5 × 10^10^ [200, 700]	6.1 × 10^10^ [50, 300]	4.2 × 10^10^ [50, 200]	3.1 × 10^10^ ≤200	1.11 × 10^11^ [50, 200]
**cryoTEM** (particle number)	369	453	316	201	744

## Data Availability

Data available on request.

## Abbreviations

EVs	Extracellular Vesicles
Cryo-TEM	Cryo-Transmission Electron Microscopy
MVB	MultiVesicular Bodies
FF	Follicular Fluid
UC	Ultracentrifugation
NTA	Nanoparticle Tracking Analysis
DLS	Dynamic Light Scattering
PBS	Phosphate-Buffered Saline
